# *LINC00673* rs11655237 C>T Polymorphism Impacts Hepatoblastoma Susceptibility in Chinese Children

**DOI:** 10.3389/fgene.2019.00506

**Published:** 2019-05-24

**Authors:** Tianyou Yang, Jiahao Li, Yang Wen, Tianbao Tan, Jiliang Yang, Jing Pan, Chao Hu, Yuxiao Yao, Jiao Zhang, Yijuan Xin, Suhong Li, Huimin Xia, Jing He, Yan Zou

**Affiliations:** ^1^Department of Pediatric Surgery, Guangzhou Women and Children’s Medical Center, Guangzhou Medical University, Guangzhou, China; ^2^The First Affiliated Hospital, Sun Yat-sen University, Guangzhou, China; ^3^Department of Pediatric Surgery, First Affiliated Hospital of Zhengzhou University, Zhengzhou, China; ^4^Clinical Laboratory Medicine Center of PLA, Xijing Hospital, Air Force Medical University, Xi’an, China; ^5^Department of Pathology, Children Hospital and Women Health Center of Shanxi, Taiyuan, China

**Keywords:** hepatoblastoma, cancer susceptibility, *LINC00673*, liver malignancy, genetic association analysis

## Abstract

**Background:**

Hepatoblastoma (HB) is the most common hepatic malignancy in children, accounting for approximately 80% of all childhood liver tumors. Previous genome-wide association studies (GWASs) have found that the *LINC00673* rs11655237 C>T polymorphism is associated with the risk of several different adult cancers. However, the association between this polymorphism and HB susceptibility remains unclear.

**Methods:**

We analyzed the association between the *LINC00673* rs11655237 C>T polymorphism and HB susceptibility in a hospital-based study of Chinese children. We enrolled 213 HB patients and 958 healthy controls with genotypes determined by TaqMan, and the strength of the association of interest was determined by calculating odds ratios (ORs) and 95% confidence intervals (CIs).

**Findings:**

We found a significant association between the *LINC00673* rs11655237 C>T polymorphism and HB risk (CT/TT compared with CC: adjusted OR = 1.40, 95% CI = 1.04–1.88, *p* = 0.029). Furthermore, stratified analysis indicated that rs11655237 T allele carriers in the following subgroups were more likely to develop HB: children older than 17 months, males, and those with tumors of clinical stages III + IV.

**Interpretation:**

In conclusion, we confirmed that the *LINC00673* rs11655237 C>T polymorphism may be associated with HB susceptibility. Prospective studies with larger sample sizes and patients of different ethnicities are needed to validate our findings.

## Introduction

Hepatoblastoma (HB) is the most common childhood hepatic malignancy, accounting for approximately 80% of all childhood liver malignancies and 1% of all childhood malignancies (von Schweinitz, 2012). HB is an embryonic tumor that may have originated from pluripotent stem cells in the liver during the embryonic period (Devi et al., 2014). Epidemiological data show that among those under 19 years of age, the incidence rate is 1.2–1.5/10^6^ individuals, with 90% of cases occurring within 3 years of birth and males being more susceptible (Herzog et al., 2000). The clinical symptoms of HB primarily consist of asymptomatic abdominal masses, which may be accompanied by fever, weight loss, anorexia, and obstructive jaundice or acute abdomen caused by tumor rupture (El Asmar and El Rassi, 2016). Alpha-fetoprotein (AFP) can be used as an important biomarker of HB: more than 90% of children with HB have elevated AFP levels, with AFP <100 ng/ml often indicating poor prognosis (Rojas et al., 2014).

The cause of HB remains unclear. It is known that unlike adult liver cancer, its occurrence is not related to hepatitis B virus (HBV) or liver cirrhosis (D’Antiga et al., 2007). Many reports suggest that HB incidence is closely related to Beckwith–Wiedemann Syndrome (BWS), familial adenomatous polyposis (FAP), glycogen storage disease, and fetal alcohol syndrome (Goldman et al., 2010; von Schweinitz, 2012). Premature birth, low body weight, neonatal radiological examination, medication, total parenteral nutrition, and other factors may also contribute to the pathogenesis of HB (Colleen et al., 2006; Hughes and Michels, 2010; El Asmar and El Rassi, 2016).

Non-coding RNAs are generally classified according to length, with those of 200–100,000 nucleotides being referred to as long non-coding RNAs (lncRNAs) (Xuefei et al., 2013). In recent years, genome-wide association studies (GWASs) have found that only 7% of single nucleotide polymorphisms (SNPs) associated with complex diseases or phenotypes are located in protein-coding regions, while the remaining 93% are located in non-coding regions (Jing et al., 2015). GWASs on cancer susceptibility have shown that some relevant SNPs are located in non-coding transcribed regions (Yang et al., 2015; Pan et al., 2016). For instance, the region upstream of the 9p21 locus (encoding the cyclin-dependent kinase inhibitors CDKN2B and CDKN2A and the P53 activator ARF) is associated with tumor susceptibility and is transcribed into lncRNA (Rivand et al., 2017). These findings suggest that genetic variation in lncRNAs may play important roles in tumorigenesis and development (Botti et al., 2018).

*LINC00673* is an lncRNA-encoding gene located on chromosome 17q24.3. It has recently been implicated as an oncogenic molecule with an important role in several physiological processes, such as cell proliferation, differentiation, and apoptosis (Shi et al., 2016; Zhang et al., 2017). Studies have found that LINC00673 plays a key role in the occurrence and development of various malignant tumors, such as pancreatic, liver, gastric, and lung cancer (Shi et al., 2016; Zheng et al., 2016; Ba et al., 2017). Moreover, studies have shown that the *LINC00673* rs7214041 polymorphism is significantly associated with the development of pancreatic cancer (Zheng et al., 2016) and neuroblastoma (Zhang et al., 2018). Zhang et al. (2017) reported that the expression of *LINC00673* was significantly elevated in liver cancer tissue and correlated with disease progression. LINC00673 may compete with miR-205 as a competing endogenous RNA, inhibiting the expression of miR-205 and promoting the development and progression of liver cancer (Zhang et al., 2017). Ba et al. (2017) reported that *LINC00673* is highly expressed in gastric cancer tissue, with expression changes being closely related to lymph node metastasis, distant metastasis, TNM staging, and gastric cancer prognosis. Knockdown of *LINC00673* expression can significantly inhibit the proliferation, migration, and invasion of gastric cancer cells (Huang et al., 2017). Ma et al. (2017) found that LINC00673 can bind to *EZH2* to inhibit *HOAX5* expression, thereby promoting the occurrence and development of non-small cell lung cancer.

Mounting evidence suggests that *LINC00673* may participate in the occurrence and development of malignant tumors in various ways. We found that *LINC00673* rs11655237 polymorphism was associated with neuroblastoma susceptibility in Chinese population (Zhang et al., 2018). However, to our knowledge, the role of *LINC00673* polymorphisms within the context of HB has not yet been reported. Therefore, in this study, we analyzed the correlation between the *LINC00673* rs11655237 C>T polymorphism and HB susceptibility.

## Materials and Methods

### Study Subjects

Hepatoblastoma patients (*n* = 213) and cancer-free controls (*n* = 958) were recruited from Guangdong, Henan, Shaanxi, and Shaanxi provinces in China for analysis (Supplementary Table S1). All 213 cases were histopathologically diagnosed with HB. These cases were genetically unrelated to the controls and matched to controls based on age, sex, and ethnicity. Each child’s parent or guardian provided written informed consent for the child’s participation in the study. This subject was approved by the Ethics Committee of Guangzhou Women and Children’s Medical Center.

### Genotyping

The hepatoblastoma DNA was extracted from paraffin section. Each subject was genotyped for the *LINC00673* rs11655237 C>T polymorphism using the TaqMan platform (Applied Biosystems, Foster City, CA, United States) (Stocker and Ishak, 1994; Hirschman et al., 2005; Rumbajan et al., 2013; He et al., 2016, 2017, 2018). Quality control was performed with positive controls and eight negative controls in each of the 384-well plates. For quality control and to verify the accuracy of the genotyping results, approximately 10% of samples were randomly selected and re-genotyped, and the concordance rate was 100%.

### Statistical Analysis

The χ^2^ test was used to evaluate differences in demographic variables, risk factors, and *LINC00673* genotype distributions between the case and control groups. The χ^2^ test was also used to determine whether the distribution of *LINC00673* genotypes was consistent with Hardy–Weinberg equilibrium (HWE). To estimate the strength of the association between the rs11655237 C>T polymorphism and HB risk, unconditional univariate and multivariate logistic regression analyses were performed, adjusting for age and sex, using odds ratios (ORs) and 95% confidence intervals (CIs). Further stratification analysis was performed based on the age, sex, and clinical stage of HB patients with different genotypes. Differences with *p*-values <0.05 were considered statistically significant. All statistical analyses were two-sided and performed using SAS software (version 9.1; SAS Institute, Cary, NC, United States).

## Results

### Characteristics of the Study Population

In this study, we enrolled 213 HB cases and 958 healthy controls. The distributions of age, sex, and clinical stages of the study subjects are shown in Supplementary Table S1. No significant differences were observed between HB patients and controls in terms of age (*p* = 0.105) or sex (*p* = 0.973). The majority of subjects in both the case and control groups were males, accounting for 60.56% (129/213) and 60.44% (579/958) of subjects, respectively. Forty-two (19.72%), 55 (25.82%), 40 (18.78%), and 15 (7.04%) patients had tumors of clinical stages I, II, III, and IV, respectively, while clinical stage was unknown in 61 cases (28.64%). The 213 HB patients and 958 healthy controls were recruited from the Guangdong, Henan, Shaanxi, and Shaanxi provinces (Supplementary Table S2). Within each province, there were no significant differences in age or sex between HB patients and healthy controls (*p* > 0.05).

### *LINC00673* rs11655237 C>T Polymorphism and HB Susceptibility

Genotype and allele frequencies of the *LINC00673* rs11655237 C>T polymorphism and associations with HB risk are summarized in Table 1. We found that carriage of the rs11655237 T allele was significantly associated with an increased risk of HB (CT compared with CC: adjusted OR = 1.41, 95% CI = 1.03–1.93, *p* = 0.031; CT/TT compared with CC adjusted OR = 1.40, 95% CI = 1.04–1.88, *p* = 0.029).

**Table 1:** *LINC00673* rs11655237 C>T polymorphism and hepatoblastoma susceptibility.

Genotype	Cases (*n* = 213)	Controls (*n* = 958)	*p*^a^	Crude OR (95% CI)	*p*	Adjusted OR (95% CI)^b^	*p*^b^
**rs11655237 (HWE, *p* = 0.232)**							
CC	115 (53.99)	595 (62.11)		1.00		1.00	
CT	85 (39.91)	312 (32.57)		1.41 (1.03–1.93)	0.031	1.41 (1.03–1.93)	0.031
TT	13 (6.10)	51 (5.32)		1.32 (0.70–2.50)	0.397	1.32 (0.69–2.50)	0.399
**Additive**			0.049	1.27 (1·00–1.62)	0.050	1.27 (1.00–1.62)	0.050
Dominant	98 (46.01)	363 (37.89)	0.028	1.40 (1·04–1.88)	0.029	1.40 (1.04–1.88)	0.029
Recessive	200 (93.90)	907 (94.68)	0.651	1.16 (0·62–2.17)	0.651	1.16 (0.62–2.17)	0.654

*^a^χ^2^ test for genotype distributions between hepatoblastoma patients and controls. ^b^Adjusted for age and sex.*

**Table 2:** Stratification analysis for the association between *LINC00673* rs11655237 C>T polymorphism and hepatoblastoma risk.

Variable	CC	CT/TT	Crude OR	*p*	Adjusted OR^a^	*p*^a^
					
	(Cases/controls)		(95% CI)		(95% CI)	
**Age, month**						
<17	65/278	49/176	1.19 (0.79–1.81)	0.411	1.19 (0.79–1.81)	0.407
≥17	50/317	49/187	1.66 (1.08–2.56)	0.022	1.67 (1.08–2.57)	0.021
**Sex**						
Female	50/235	34/144	1.11 (0.69–1.80)	0.672	1.08 (0.67–1.76)	0.748
Male	65/360	64/219	1.62 (1.10–2.38)	0.014	1.61 (1.10–2.37)	0.015
**Clinical stage**						
I+II	52/595	45/363	1.42 (0.93–2.16)	0.103	1.42 (0.94–2.17)	0.099
III+IV	26/595	29/363	1.83 (1.06–3.15)	0.030	1.84 (1.07–3.17)	0.029

*OR, odds ratio; CI, confidence interval. ^a^Adjusted for age and sex, omitting the corresponding stratification factor.*

### Stratification Analysis of Association Between *LINC00673* rs11655237 C>T Polymorphism and HB Risk

To further evaluate the effect of the *LINC00673* rs11655237 C>T polymorphism on HB risk, stratification analysis was further used to evaluate the association between the *LINC00673* rs11655237 C>T polymorphism and HB risk in different strata (based on age, sex, and clinical stage) (Table 2). Significant associations was detected in the following subgroups: children older than 17 months (adjusted OR = 1.67; 95% CI = 1.08–2.57, *p* = 0.021), males (adjusted OR = 1.61; 95% CI = 1.10–2.37, *p* = 0.015), and patients with tumors of clinical stages III + IV (adjusted OR = 1.84, 95% CI = 1.07–3.17, *p* = 0.029).

## Discussion

The relationship between the *LINC00673* rs11655237 C>T polymorphism and the risk of HB was investigated in this hospital-based case-control study. The results of our study showed that the *LINC00673* rs11655237 C>T polymorphism was significantly associated with susceptibility to HB. Furthermore, stratified analysis indicated that rs11655237 T allele carriers in the following subgroups were more likely to develop HB: children older than 17 months, males, and patients with tumors of clinical stages III + IV. To our knowledge, we are the first to confirm that the *LINC00673* rs11655237 C>T polymorphism is associated with HB susceptibility.

HB is an embryonic tumor that ranks first among childhood liver tumors and seriously endangers children’s health (Herzog et al., 2000). HB and hepatocellular carcinoma are characterized by differences in incidence, age distribution, sex distribution, birth history, genetic basis, and related risk factors (Jeng et al., 2000). Rumbajan et al. (2013) and (Udatsu et al., 2001) showed that in HB patients, some differentially methylated regions (DMRs) exhibited abnormal methylation prior to the development of HB, suggesting that changes in the methylation of DMRs are related to the occurrence of HB. Moreover, studies have shown that the inactivation of the tumor suppressor gene adenomatous polyposis coli (*APC*), the main function of which is the downregulation of β-catenin, is involved in the occurrence of HB (Li et al., 2014). Indeed, some researchers have used β-catenin as an indicator for evaluating the prognosis of HB (Froberg et al., 2013; Pickard and Williams, 2015).

Over the past decade, it became clear that lncRNAs play an important regulatory role in various processes, including metastasis, imprinting, tumor suppressor dysregulation, and X inactivation (Childs et al., 2015; Gao and Wei, 2017; Yu et al., 2017). In addition, aberrant expression and polymorphisms of lncRNAs are associated with susceptibility to a range of human diseases, including cancer, and these can represent new targets for the diagnosis and treatment of cancer (Amundadottir, 2016). *LINC00673* may also act as a tumor suppressor by promoting interaction between protein tyrosine phosphatase, non-receptor type 11 (Ptpn11) and ubiquitin ligase, resulting in degradation of Ptpn11 and lowered oncogenic signaling (Huang et al., 2017). *LINC00673* has been shown to be involved in susceptibility to and progression and outcome of many malignancies, acting as either a tumor suppressor or promoter (Wang and Luo, 2018). The relationship between *LINC00673* rs11655237 and pancreatic cancer susceptibility in individuals of European descent was identified through GWAS of 9,925 cases of pancreatic cancer and 11,569 controls (Childs et al., 2015). Meanwhile, Zheng et al. (2016) replicated these findings in the Chinese population and found that rs11655237 produced a miR-1231-binding site and interfered with the degradation of PTPN11. Wang and Luo (2018) revealed that the *LINC00673* rs11655237 C>T polymorphism is associated with an increased risk of cervical cancer, possibly by downregulating *LINC00673* expression in cervical tissues. Zhang et al. (2018) verified that the *LINC00673* rs11655237 C>T polymorphism may be associated with neuroblastoma susceptibility.

In this study, 213 HB patients and 958 cancer-free controls from four different provinces across China were genotyped to evaluate the association between the *LINC00673* rs11655237 C>T polymorphism and the risk of HB. Our results showed that the *LINC00673* rs11655237 C>T polymorphism may indeed affect HB susceptibility. However, HB is a multifactorial disease that may also be affected by environmental factors and genetic background. Our research is limited by the lack of valuable information on these other aspects, including dietary intake and the living environment of the parents. Selection bias is another obvious possible confounding factor, and the study population should not be considered to represent the entire Chinese population. Moreover, this study investigated only one polymorphism, and other polymorphisms of *LINC00673*, alone or in combination, should be investigated. Finally, environmental factors that may interact with the *LINC00673* polymorphism were not investigated. The relationship between this polymorphism and patients outcome was not investigated, due to insufficient outcome data. To better elucidate the relationship of the *LINC00673* polymorphism with susceptibility to HB, future studies should be designed to avoid these shortcomings as much as possible. The expression of *LINC00673* may need to be tested.

## Conclusion

In conclusion, our study is the first to analyze the correlation between the *LINC00673* rs11655237 C>T polymorphism and HB risk among the Chinese population. Our results confirmed that the *LINC00673* rs11655237 C>T polymorphism may have significant effects on HB risk in the Chinese population.

## Ethics Statement

Each child’s parent or guardian provided written informed consent for the child’s participation in the study. This subject was approved by the Ethics Committee of Guangzhou Women and Children’s Medical Center.

## Author Contributions

TY, JL, JZ, YX, SL, and HX conceptualized and designed the study, drafted the initial manuscript, and reviewed and revised the manuscript. YW, TT, JY, JP, CH, and YY designed the data collection instruments, collected data, carried out the initial analyses, and reviewed and revised the manuscript. YZ and JH conceptualized and designed the study, coordinated and supervised data collection, and critically reviewed the manuscript for important intellectual content. All authors approved the final manuscript as submitted and agreed to be accountable for all aspects of the work.

## Conflict of Interest Statement

The authors declare that the research was conducted in the absence of any commercial or financial relationships that could be construed as a potential conflict of interest.
