# Supplementary material for: LINC00673 rs11655237 C>T Polymorphism Impacts Hepatoblastoma Susceptibility in Chinese Children
Source: Front Genet. 2019 May 24;10:506. doi: 10.3389/fgene.2019.00506 (PMC6544040; doi:10.3389/fgene.2019.00506)
Supplement: Supplementary file 1 [file Table_1.DOCX]

| **Supplemental Table 1**. Frequency distribution of selected variables in hepatoblastoma patients and controls | | | | | |
| --- | --- | --- | --- | --- | --- |
| Variable | Cases (n=213) | | Controls (n=958) | | p ^a^ |
|  | No. | % | No. | % |  |
| Age range, months | 0·23-149·97 | | 0·004-156·00 | | 0·105 |
| Mean ± SD | 23·62 ± 24·36 | | 23·75 ± 18·30 | |  |
| <17 | 114 | 53·52 | 454 | 47·39 |  |
| ≥17 | 99 | 46·48 | 504 | 52·61 |  |
| Sex |  |  |  |  | 0·973 |
| Female | 84 | 39·44 | 379 | 39·56 |  |
| Male | 129 | 60·56 | 579 | 60·44 |  |
| Clinical stage |  |  |  |  |  |
| I | 42 | 19·72 |  |  |  |
| II | 55 | 25·82 |  |  |  |
| III | 40 | 18·78 |  |  |  |
| IV | 15 | 7·04 |  |  |  |
| NA | 61 | 28·64 |  |  |  |
| ^a^ Two-sided *χ^2^* test for distributions between hepatoblastoma patients and cancer-free controls. | | | | | |

| **Supplemental Table 2**. Frequency distribution of selected variables in hepatoblastoma patients and controls | | | | | | | | | | | | |
| --- | --- | --- | --- | --- | --- | --- | --- | --- | --- | --- | --- | --- |
| Variable | Guangdong province | | | Henan province | | | Shaanxi province | | | Shanxi province | | |
|  | Cases (n=146) | Controls (n=438) | p ^a^ | Cases (n=42) | Controls (n=176) | p ^a^ | Cases (n=15) | Controls (n=186) | p ^a^ | Cases (n=10) | Controls (n=158) | p ^a^ |
|  | No. (%) | No. (%) |  | No. (%) | No. (%) |  | No. (%) | No. (%) |  | No. (%) | No. (%) |  |
| Age range, months | 0·63-149·97 | 0·07-156·00 | 0·214 | 0·83-108·00 | 0·10-108·00 | 0·285 | 3·60-72·00 | 0·03-60·00 | 0·286 | 0·23-72·00 | 0·004-60·00 | 0·785 |
| Mean ± SD | 23·16±24·59 | 23·11±18·62 |  | 26·73±24·96 | 27·28±18·87 |  | 21·50±24·20 | 23·66±16·66 |  | 20·32±20·38 | 21·70±18·28 |  |
| <17 | 79 (54·11) | 211 (48·17) |  | 21 (50·00) | 72 (40·91) |  | 9 (60·00) | 85 (45·70) |  | 5 (50·00) | 86 (54·43) |  |
| ≥17 | 67 (45·89) | 227 (51·83) |  | 21 (50·00) | 104 (59·09) |  | 6 (40·00) | 101 (54·30) |  | 5 (50·00) | 72 (45·57) |  |
| Sex |  |  | 0·961 |  |  | 0·830 |  |  | 0·544 |  |  | 0·912 |
| Female | 58 (39·73) | 175 (39·95) |  | 15 (35·71) | 66 (37·50) |  | 7 (46·67) | 72 (38·71) |  | 4 (40·00) | 66 (41·77) |  |
| Male | 88 (60·27) | 263 (60·05) |  | 27 (64·29) | 110 (62·50) |  | 8 (53·33) | 114 (61·29) |  | 6 (60·00) | 92 (58·23) |  |
| Clinical stage |  |  |  |  |  |  |  |  |  |  |  |  |
| I | 6 (4·11) | / |  | 19 (45·24) | / |  | 15 (100·00) | / |  | 2 (20·00) | / |  |
| II | 46 (31·51) | / |  | 3 (7·14) | / |  | / | / |  | 6 (60·00) | / |  |
| III | 37 (35·34) | / |  | 3 (7·14) | / |  | / | / |  | 0 (0·00) | / |  |
| IV | 12 (8·22) | / |  | 1 (2·38) | / |  | / | / |  | 2 (20·00) | / |  |
| NA | 45 (30·82) | / |  | 16 (38·10) | / |  | / | / |  | / | / |  |
| ^a^ Two-sided *χ^2^* test for distributions between hepatoblastoma patients and cancer-free controls. | | | | | | | | | | | | |
